# *Staphylococcus epidermidis* Has Growth Phase Dependent Affinity for Fibrinogen and Resulting Fibrin Clot Elasticity

**DOI:** 10.3389/fmicb.2021.649534

**Published:** 2021-06-16

**Authors:** Carolyn Vitale, Tianhui Maria Ma, Janice Sim, Christopher Altheim, Erika Martinez-Nieves, Usha Kadiyala, Michael J. Solomon, J. Scott VanEpps

**Affiliations:** ^1^Department of Pediatric Cardiology, University of Michigan, Ann Arbor, MI, United States; ^2^Department of Chemical Engineering, University of Michigan, Ann Arbor, MI, United States; ^3^Department of Emergency Medicine, University of Michigan, Ann Arbor, MI, United States; ^4^Biointerfaces Institute, University of Michigan, Ann Arbor, MI, United States; ^5^Department of Biomedical Engineering, University of Michigan, Ann Arbor, MI, United States; ^6^Michigan Center for Integrative Research in Critical Care, University of Michigan, Ann Arbor, MI, United States; ^7^Macromolecular Science and Engineering, University of Michigan, Ann Arbor, MI, United States

**Keywords:** bacterial adhesion, biofilm, *SdrG*, *SSPA*, rheometry, thrombosis, virulence

## Abstract

Bacterial infection and thrombosis are highly correlated, especially in patients with indwelling medical devices. Coagulase-negative staphylococci, typified by *Staphylococcus epidermidis*, are a common cause of medical device infections owing to their biofilm forming capacity which provides protection from antibiotics and host immune response. Attention has been drawn to the interaction between *S. epidermidis* and host proteins, specifically fibrinogen. However, little is known regarding the impact of the transition from planktonic to biofilm forming phenotype on this interaction. Here we investigate the growth phase dependence of bacteria-fibrinogen interaction and the resulting effect on fibrin clot formation, structure, and mechanics. Flow cytometry demonstrated growth phase dependent affinity for fibrinogen. To mimic intravascular device seeding, we quantified the adhesion of *S. epidermidis* to a fibrinogen coated surface under continuous flow conditions *in vitro*. The bacterial deposition rate onto fibrinogen was significantly greater for stationary (5,360 ± 1,776 cells/cm^2^s) versus exponential phase (2,212 ± 264, cells/cm^2^ s). Furthermore, the expression of *sdrG*–a cell surface adhesion protein with specificity for fibrinogen–was upregulated ∼twofold in the stationary versus the exponential phase. Rheometry and confocal microscopy demonstrated that stationary phase *S. epidermidis* slows clot formation and generates a more heterogeneous fibrin network structure with greater elasticity (*G*′ = 5.7 ± 1.0 Pa) compared to sterile fibrinogen (*G*′ = l.5 ± 0.2 Pa), while exponential phase cells had little effect. This work contributes to the current understanding of the growth phase dependent regulation of bacterial virulence factors and the correlation between bacterial infection and thrombosis.

## Introduction

Pathologic clots, for example, venous thromboembolism and embolic cerebrovascular accidents, are highly correlated with bacterial infection (Raad et al., 1994; Grau et al., 1995; Samama, 2000; Dalager-Pedersen et al., 2014). Clinically, thrombosis and bacterial biofilms often appear simultaneously, particularly on implanted medical devices (Raad et al., 1994; Brackman et al., 2013). One study showed that 12 of 14 patients with central venous catheter (CVC) related infection had a preceding CVC related venous thrombosis (Lordick et al., 2003) and a relative risk of thrombosis is assigned to the presence of infection (VanEpps and Younger, 2016). One of the mechanisms to explain such correlation is that clotting proteins provide a substrate for bacterial adherence with microbial receptor-specific binding to fibrinogen and its cleaved product fibrin. Specifically, *Staphylococcus epidermidis* has a well described cell wall anchored (CWA) surface protein called *SdrG* which binds fibrinogen in a “dock, lock, and latch” mechanism (Nilsson et al., 1998; Davis et al., 2001; Hartford et al., 2001; Ponnuraj et al., 2003; Bowden et al., 2005, 2008; Foster, 2020). *SdrG* binding to fibrinogen is strengthened under conditions of shear stress (e.g., fluid flow like that seen around vascular catheters) (Weaver et al., 2011; Milles et al., 2018) and is extremely strong, approaching that of a covalent bond (Herman et al., 2014). Although it appears that *SdrG* binding is sensitive to forces generated under typical centrifugation conditions and highly strain dependent (Vanzieleghem et al., 2015). Despite this potential complexity in binding activity, antibodies against this CWA surface protein block *S. epidermidis* interactions with peripheral venous catheters (Pei and Flock, 2001) and diminished CVC associated infection and resulting bacteremia in mice (Guo et al., 2007). In addition, *SdrG* promotes platelet adhesion and aggregation *via* interaction with fibrinogen (Brennan et al., 2009). Therefore, *SdrG* is considered a critical mediator of the interaction between *S. epidermidis* infection and thrombosis.

As compared to *Staphylococcus aureus*, which has many more virulence factors, *S. epidermidis* relies mostly on its ability to form biofilms to cause infection (Becker et al., 2014). A biofilm is a bacterial community that occurs when bacteria attach to a surface, alter their phenotype and produce a protective layer of extracellular polymeric substance (EPS). A significant cause of clinical infections, biofilms present a profound obstacle to host immune defense, mechanical debridement, and treatment with traditional antimicrobials (Otto, 2009, 2018). The most commonly recognized biofilm infections are those related to indwelling medical devices (Wolfmeier et al., 2018). The life cycle of a biofilm (reviewed in VanEpps and Younger, 2016; Otto, 2018) begins when bacteria adhere to a surface followed by an initial period of exponential growth and EPS production. Once the biofilm becomes densely populated and encased in EPS, the growth slows. Finally, some of the bacteria detach as planktonic (i.e., free floating) or clusters (i.e., flocs) of bacteria that can attach to a new substrate elsewhere and develop another biofilm. Each phase of the life cycle requires specific protein machinery to be most effective and the bacteria display phenotypic changes as they progress from planktonic to biofilm phenotype. While the change in phenotype is key in biofilm formation, little is known about the impact of growth phase on bacteria-host protein interaction, specifically fibrinogen. In fact, all of the prior literature evaluating *S. epidermidis* binding to fibrinogen focuses specifically on cells in the stationary phase of growth.

*Staphylococcus epidermidis*, a ubiquitous, commensal colonizer of human skin, belongs to the group of coagulase negative staphylococcal species which are the leading cause of device related infection, endocarditis and surgical site infections (Rogers et al., 2009; Gahlot et al., 2014). Device related infections, particularly central line associated bloodstream infections increase mortality, medical cost and hospital length of stay (Stevens et al., 2014; Ziegler et al., 2014). Previous work from our group showed that not only does *S. epidermidis* adhere to fibrin clots, its presence also influences the clot microstructure and mechanics in a way that has implications for bacterial induced thromboembolism (Ma et al., 2017).

The goal of this work is to explore the phenotypic differences between stationary and exponential phase *S. epidermidis* in the scope of their interactions with fibrinogen and their impact on fibrin network formation, structure and mechanics. We first applied flow cytometry to characterize the growth-phase dependent bacterial affinity for fibrinogen. Understanding that force/shear loading affect fibrinogen binding, we then evaluated phase dependent *S. epidermidis* surface deposition onto fibrinogen in a continuously flowing model mimicking seeding of an intravascular device. Next, we compared phase differences in gene expression of *S. epidermidis* cell surface proteins with potential impact on adhesion and interaction with host coagulation proteins. Finally, we performed rheology and confocal microscopy to evaluate the structure and mechanics of exponential versus stationary phase *S. epidermidis* infected fibrin clots.

## Materials and Methods

### Bacterial Strains and Culture

*Staphylococcus epidermidis* strain RP62A, a pathogenic strain which forms biofilms, was used for all experiments. Loop inoculates from frozen glycerol stock (–80°C) were grown on tryptic soy agar (TSA, Fisher Scientific) plates for at least 16 h at 37°C. A single colony was selected and cultured in tryptic soy broth with glucose 1% (TSBG) at 37°C under agitation at 200–250 rpm. Exponential phase growth was defined as an optical density of 0.4 ± 0.1 measured at 600 nm (Supplementary Figure 1). A 30 mL sample of the culture at this phase was removed and left undisturbed for 30–60 min at room temperature to allow for settling of flocculated cells prior to use in subsequent studies. For stationary phase, the remaining culture was returned to the incubator at 37°C to continue growth under agitation until an optical density of 1.4 ± 0.05 was obtained. Since rheometry experiments required significantly larger volumes, separate cultures were grown to exponential and stationary phases as defined above.

### Quantification of Fibrinogen Affinity by Flow Cytometry

*Staphylococcus epidermidis* were cultured to exponential and stationary phase as described above. Cell density was quantified in each sample with a Incyto Hemacytometer (Thermo Fisher Scientific, Waltham, MA, United States) and cells were centrifuged into a pellet at 5,000 rpm for 3 min. Supernatant was discarded, and pelleted cells were diluted to concentration of 2.0 × 10^9^ cells/mL. Two conditions were prepared in triplicate for each growth phase–unlabeled control and labeled fibrinogen. Control and labeled fibrinogen pellets were resuspended in 1X Hank’s Buffered Salt Solution (with Calcium, Magnesium, no phenol red, 1 g/L D-Glucose, Thermo Fisher Scientific, Waltham, MA, United States). Fluorescently labeled fibrinogen (Alexafluor 647 Fibrinogen, 650/668 nm, Thermo Fisher Scientific, Waltham, MA, United States) was added to a final concentration of 0.5 mg/mL and incubated for 15 min. Cells were then analyzed by flow cytometry (Cytoflex, Beckman). Histograms of area fluorescence (excitation 650 nm/emission 668 nm) from 10^5^ events per sample were obtained. Mean fluorescence intensity (MFI) was determined as a surrogate for total fibrinogen concentration on the cell surface using FCS Express (v.7, De Novo Software, Pasadena, CA, United States). Nine experiments were completed in triplicate with the median and interquartile range (IQR) reported. Statistical analyses were performed with Wilcoxon paired signed rank test with significance defined as *p* < 0.05.

### Bacterial Adhesion to Fibrinogen in Continuously Flowing Environment

Fibrinogen coating of glass coverslips was performed as previously described (Kuusela et al., 1985) with minor modification. Briefly, fibrinogen from human plasma (EMD Millipore Corporation, Billerica, MA, United States) was reconstituted as directed and stored at –80°C in aliquots. Frozen aliquots were thawed and diluted to concentration of 10 μg/mL using sterile distilled water. Glass coverslips were coated with fibrinogen for 4 h at 20°C, blocked with 1% bovine serum albumin for 1 h, and washed three times with normal saline. This method was previously determined to have ∼80% binding efficiency (Kuusela et al., 1985) and for our geometry is estimated to result in a fibrinogen surface density of 80 molecules/μm^2^.

The stationary phase sample was diluted with normal saline to match optical density of the exponential phase culture then left undisturbed at 20°C for 1 h to remove large flocculates. Exponential and stationary phase cells were fluorescently labeled with Syto 9 (Thermo Fisher Scientific, Waltham, MA, United States). Cells were loaded into a 10 mL syringe on a standard syringe pump (Harvard Apparatus). Fibrinogen coated glass coverslips were assembled into a parallel-plate flow chamber (Warner instruments RC-31 with 250 μm thick, 3.2 mm slot gasket). Cell suspensions were infused at a rate of 125 μL/min (Reynolds number = 1.2, wall shear stress = 0.067 Pa) through the parallel-plate flow chamber mounted on a epifluorescent microscope (Nikon Eclipse 80i). Images were captured every 3 s for 30 min. A total of 10 experiments were completed in duplicate and the mean for each experiment was used.

Custom image analysis was performed using MATLAB (v.R2018b, Natick, MA, United States). Briefly, a new adhesion event was defined as the new appearance of a bacterium which persisted for two or more consecutive images in a single location. The deposition rate was calculated as the slope of adhesion events versus time for each experimental replicate. Residence time was defined as the total time a single bacterium persisted at a single location. Student’s paired *t*-test was used for hypothesis testing comparing deposition rate, expressed as cells/cm^2^ s, versus growth phase with *p* < 0.05 defined as significant. For residence time in seconds, a χ^2^ test was used to compare the population of bacteria persisting for greater than 500 s for each growth phase with significance defined as *p* < 0.01.

### Gene Expression

Target genes were selected to probe for proteins whose function may be responsible for the observed differences in bacterial binding to fibrinogen. Specifically, we chose genes involved in bacterial adhesion ranging from cell–cell adhesion *via aap* and *icaA*, cell-abiotic adhesion *via atlE* and cell-host protein (e.g., fibrin/fibrinogen, collagen) adhesion *via* the *sdr* family. In addition, *sspA*, a serine protease which putatively degrades fibrinogen (Otto, 2009; Sugimoto et al., 2013), was probed as a potential mediator of bacterial release from and/or subsequent degradation of the fibrin clot. The *ica* repressor gene (*icaR*) was probed as a control as this gene has been shown to decrease in the transition from exponential to stationary phase (Yao et al., 2005).

RNA was extracted from *S. epidermidis* grown to either exponential or stationary phase. One mL of bacterial cultures was centrifuged at 8,000 rpm for 1 min to pellet the cells. After washing with RNase free water, cells were resuspended. Cell lysis was achieved with 5 μL lysostaphin (2 μg/μL), incubation at 37°C for 45 min followed by addition of 75 μL of TRIzol and vigorous pipetting. Samples were vortexed and then centrifuged at 12,000 rpm for 10 min at 4°C. Supernatant was removed and incubated at room temperature for 5 min. Chloroform (15 μL) was added to the sample, vortexed for 15 s, and incubated at room temperature for 3 min. Following this, samples were centrifuged at 12,000 rpm for 15 min at 4°C. The aqueous phase was isolated and transferred to RNeasy Mini Kit spin columns (Qiagen) and RNA was purified per the manufacturer’s instructions. High quality RNA yield was confirmed by UV spectrophotometry (NanoDrop 2000, Thermo Scientific) and RNA integrity was confirm using a Bioanalyzer (Agilent 2100). Samples were stored at –80°C prior to RT-PCR.

QuantiTect Probe RT-PCR (Qiagen) was performed in 50 μL reactions using an Applied Biosystems Step 1+ real time PCR system. The reaction mixture contained 25 μL 2 × Probe RT-PCR Master Mix, 0.5 μL RT Mix, 5 μL of exponential or stationary phase RNA template (5 ng), 5 μL of each gene forward/reverse-primer (F/R-primer, 1 μM) and probe (0.2 μM) and 4.5 μL of RNase-free water. The RT-PCR Master Mix contained HotStarTaq DNA polymerase, ROX reference dye, dNTP mix, and RT-PCR buffer [*Tris*–HCl, KCl, (NH_4_)SO_4_, 8 mM MgCl_2_, at pH 8.7]. Thermal cycling protocol began with 30 min reverse transcription step at 50°C, followed by an initial activation step of 15 min at 95°C, denaturation of 15 s at 94°C, and combined annealing/extension step at 65°C for 60 s for a total of 45 cycles. Sequences for forward, reverse and probe oligonucleotides are shown in Supplementary Table 1. Fold change in expression (stationary vs. exponential phase) was determined using the ΔΔCT method with the 16s ribosomal subunit as an endogenous control. Student’s *t*-test was used for hypothesis testing of significance (*p* < 0.05) of the fold change in gene expression. *p*-values were corrected for multiple comparisons using Benjamin-Hochberg control of false-discovery rate.

### Rheometry and Microstructure Measurements of Fibrin Clots

Materials and constitution methods are described in detail in previously (Ma et al., 2017). Briefly, citrate-free thrombin from human plasma and fibrinogen (Fg) from human plasma were purchased from EMD Millipore (Billerica, MA, United States). Fibrinogen solutions (0.5 mg/mL) were made by diluting the stock solution with 1X Hank’s Balanced Salt Solution at 37°C. Clot was initiated by adding thrombin (0.5 U/mL as final concentration). Clot elasticity was characterized by measuring the linear elastic modulus, *G*′, on a mechanical rheometer (TA Instruments AR-G2, cone and plate fixture with a 2° cone angle and a 40 mm diameter). All tests were performed at a frequency *ω* = 0.1 Hz under an imposed strain γ = 0.01 at 37°C in a humid atmosphere. Clot microstructures were observed using a Nikon A1Rsi confocal laser scanning microscope with a 100 ×, 1.45 NA, oil immersion objective and images were analyzed to determine mesh size using MATLAB as we have previously described (Ma et al., 2017). The steady state of fibrin structures is defined as the variability in *G*′ in any 2 min interval being no more than 0.5% for 20 min (Ma et al., 2017). Bacteria concentration for both exponential and stationary phase was 4.0 × 10^9^ cells/mL to match *in vivo* biofilm concentrations (Lungren et al., 2014). Statistical comparisons were made *via* ANOVA with post-hoc pair-wise Tukey test with significance level of *p* < 0.05.

## Results

### Affinity for Fibrinogen

**FIGURE 1:**
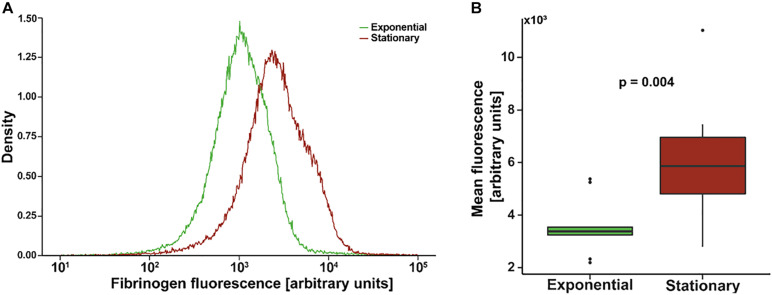
Flow cytometry of *Staphylococcus epidermidis* incubated with fluorescently labeled fibrinogen. **(A)** Example density plot of fibrinogen fluorescence for a single flow cytometry experiment. **(B)** Standard box (IQR) and whiskers (1.5*IQR) plot of mean fluorescence of *S. epidermidis* in exponential versus stationary phase. Bacterial cell concentration was 4 × 10^9^ cells/mL and fibrinogen concentration was 0.5 mg/mL. *N* = 9 paired experiments with triplicate measurements.

Figure 1A shows an example of a single flow cytometry experiment to quantify fibrinogen affinity. The mean fluorescence of the stationary phase population is greater than that of the exponential phase with a similar distribution. Composite mean fluorescence index of all experiments (*N* = 9 with triplicate measures) is shown in Figure 1B. On average, the mean fluorescence of stationary *S. epidermidis* incubated with fluorescently labeled fibrinogen was significantly higher as compared to exponential phase under the same condition (3,642 ± 349 vs. 6,388 ± 919, *p* = 0.004).

### Adhesion to Fibrinogen Coated Surface

**FIGURE 2:**
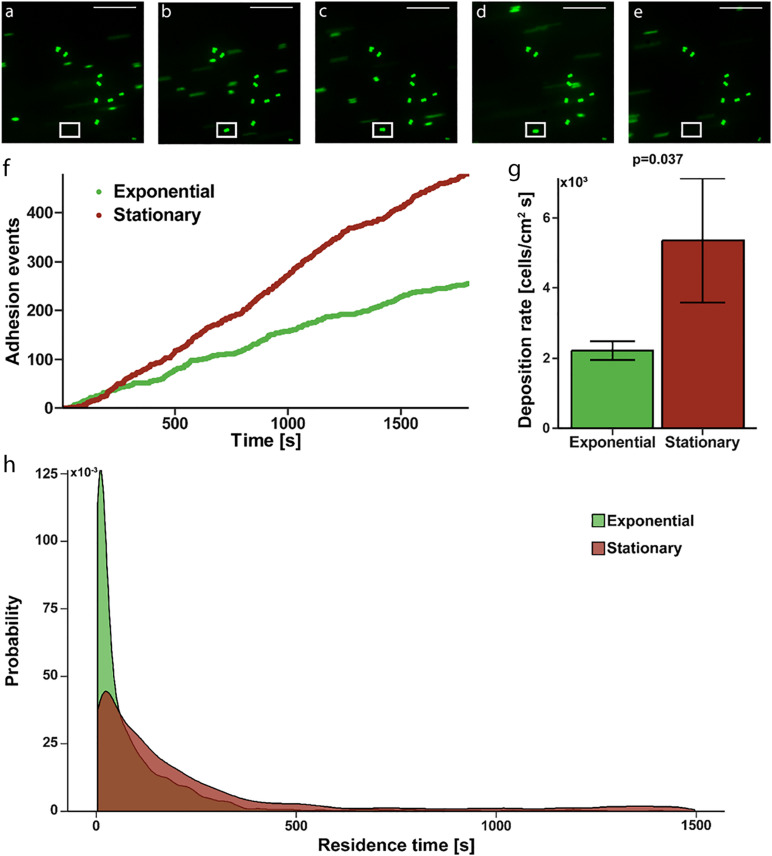
Adhesion of *S. epidermidis* to a fibrinogen coated surface under continuous flow. **(A–E)** Representative sequential images of fluorescently labeled *S. epidermidis* (green) captured every 3 s with absence of a cell in white frame in panel **(A)**, adhesion event demonstrated in frames **(B–D)** and subsequent absence in panel **(E)** representing a release event. Time between frames **(B–E)** represents the residence time of that single cell. Scale bar = 20μm. **(F)** Graphical representation of a single experiment of adhesion events over time for exponential (green) versus stationary phase (red). **(G)** Deposition rate of exponential versus stationary phase *S. epidermidis* for *N* = 10 paired experiments with duplicate measures. **(H)** Residence time probability distribution of exponential versus stationary phase *S. epidermidis* for all 10 paired experiments with duplicate measures.

To determine if the increased affinity translated to actual adhesion to a fibrinogen coated surface, where shear induced binding enhancement may contribute, we evaluated deposition rate and residence time in a continuous flow, parallel-plate system. Fluorescently labeled *S. epidermidis* flowing past immobilized fibrinogen appear as streaks on image capture but are more clearly defined circular shapes as they slow and come to rest on the surface (Figures 2A–E). Persistence of a cell in a single location for more than one consecutive image defined an adhesion event (white box in Figures 2B–D). Absence of a previously identified adhered bacteria (white box in Figure 2E) defined a release event. The total time spent adhered was defined as the residence time. The number of adhesion events in a high power field of *S. epidermidis* on immobilized fibrinogen showed a steady, linear increase over time for both exponential and stationary phase (Figure 2F); however, the deposition rate (slope of adhesion events vs. time) of stationary phase cells was significantly greater than the deposition rate of cells in exponential phase (5,360 ± 1,776 cells/cm^2^ s vs. 2,212 ± 264, cells/cm^2^ s, *p* = 0.037) (Figure 2G). Additionally, stationary phase *S. epidermidis* had longer residence time when compared to exponential phase (Figure 2H). Most notably there was a population of stationary phase cells that had a residence time of greater than 500 s, skewing the distribution to the right as compared to the exponential phase. That is, only 2.7% of exponential cells had a residence time of greater than 500 s while 13.1% of the stationary phase cell had a residence time greater than 500 s (*p* = 0.004).

### Gene Expression

To identify potential mediators of growth phase-dependent fibrinogen adhesion, phase dependent changes in expression of specific adhesin genes were determined. The relative fold-change in gene expression for stationary phase compared to exponential phase *S. epidermidis* are shown in Table 1. There was a modest, 2.2-fold increase in *sdrG* expression. Other cell adhesion molecules had no significant change in expression under these conditions. We also considered the serine protease, *sspA*, which has been predicted to specifically degrade fibrinogen (Otto, 2009; Sugimoto et al., 2013). There was a 3.5-fold increase in *sspA* expression in the stationary versus exponential phase. As predicted, *icaR* showed a decrease in expression in the transition from exponential to stationary phase.

**TABLE 1:** Fold change in gene expression for stationary (Sp) versus exponential phase (Ep).

Gene	**Function**	**Fold Change (Sp/Ep)**	**95% CI**	Corrected *p*-value*
*aap*	Cell-cell adhesion	2.1	1.00–3.20	0.076
*atlE*	Cell adhesion to abiotic surfaces	1.1	0.45–1.75	0.696
*icaA*	Peptidoglycan synthesis, cell-cell adhesion	1.6	0.75–2.35	0.175
*icaR*	**Repressor for *ica* operon**	**0.7**	**0.55–0.85**	**0.045**
*sdrF*	Adhesion to collagen	1.2	0.37–2.03	0.653
*sdrG*	**Adhesion to fibrin/fibrinogen**	**2.2**	**1.29–3.11**	**0.047**
*sspA*	**Serine Protease, degrades fibrinogen**	**3.5**	**1.53–5.47**	**0.047**

*p-values corrected for multiple comparisons using Benjamin–Hochberg control of False-Discovery Rate. Bold rows indicate statistically significant changes in expression.

### Mechanics and Structure of *S. epidermidis* Infected Fibrin Clots

**FIGURE 3:**
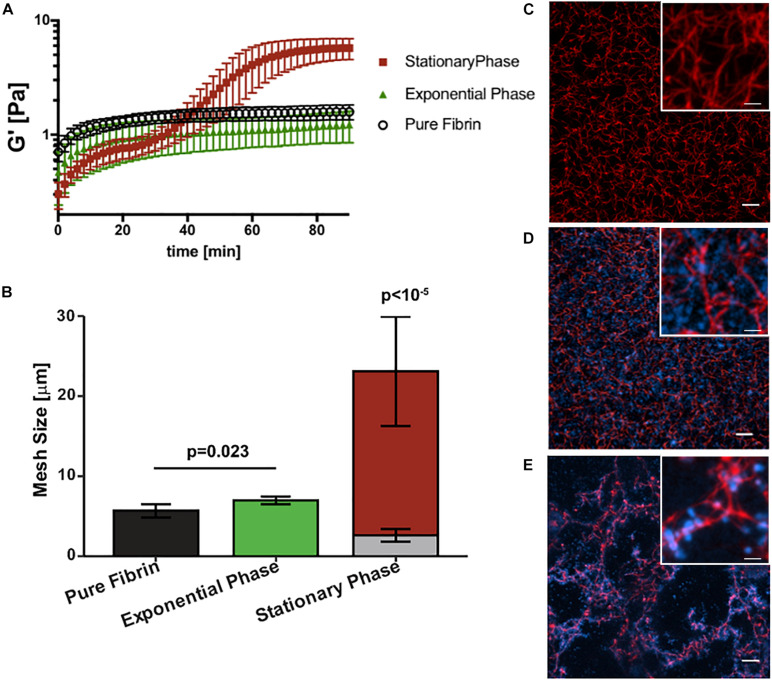
*Staphylococcus epidermidis* alters fibrin elasticity and structure in a growth phase dependent manner. **(A)** Time sweep of elastic modulus, *G*′, of pure fibrin clot (0.5 mg/mL) (black) and fibrin clots infected by stationary and (red), exponential phase *S. epidermidis* (green). Error bars denote standard deviation with *N* = 3. **(B)** Mesh size characterization of steady-state pure fibrin clot and fibrin clots infected with stationary and exponential phase *S. epidermidis*. Error bars denote standard deviations with *N* = 5. Note that the stack bars (gray and red) in the stationary phase condition indicate two characteristic mesh sizes. **(C)** Axial projections of a pure fibrin clot, a fibrin clot infected with **(D)** exponential and **(E)** stationary phase *S. epidermidis*. Scale bar = 10 m. Insets with higher magnification, scale bars = 2.5 μm. Fibrin is red and cells are blue. In all cases, cell concentrations are all 4 × 10^9^ cells/mL and fibrin concentration is 0.5 mg/mL.

Next we determined how growth phase-dependent fibrinogen binding affects fibrin clot mechanics and structure. Clot formation during rheometry is observed by rapid increase in the linear elastic modulus (*G*′) over time (Figure 3A). Based on our previous work (Ma et al., 2017) and reproduced here for purposes of comparison, the *G*′ of a pure fibrin clot (0.5 mg/mL fibrinogen) without bacterial cells reaches a steady-state at about 40 min with a value of 1.5 ± 0.2 Pa. The addition of stationary phase *S. epidermidis* retards clot formation with a two-step kinetic profile of *G*′ (Figure 3A), consistent with our previous work (Ma et al., 2017). The first step represents a transient plateau at a lower elastic modulus than the pure fibrin clot. This plateau is followed by a second rapid increase in *G*′ to a final steady-state value of 5.7 ± 1.0 Pa. This two-step kinetic profile of clot formation is completely absent for exponential phase *S. epidermidis* of the same cell concentration. In addition, the steady state for this condition is not reached within 100 min. The growth of the elastic modulus of the exponential phase is significantly retarded relative to the stationary phase. Its kinetics are also somewhat slower than pure fibrin. The final (*t* = 100 min) modulus of the exponential phase is not significantly lower than that of pure fibrin (1.3 ± 0.4 Pa).

Figures 3B–E compare the steady-state structures of the corresponding conditions with the fibrin elasticity measurements. The pure fibrin network has a homogeneous pore size distribution. As we have previously shown (Ma et al., 2017) and reproduced here for purposes of comparison, the pure fibrin mesh size under these conditions is 5.7 ± 0.8 μm (Figure 3C). This value is consistent with other measurements in the literature (Piechocka et al., 2010). The fibrin network structure formed in the presence of exponential phase *S. epidermidis* remains homogeneous (Figure 3D) and the mesh size is slightly increased (7.0 ± 0.5 μm) over pure fibrin (*p* = 0.023). In comparison, the network formed in the presence of stationary phase *S. epidermidis* is heterogeneous (Figure 3E) with two characteristic mesh sizes (2.6 ± 0.8 and 23.1 ± 6.8 μm), as previously reported (Ma et al., 2017), which are significantly different than either pure fibrin or fibrin with exponential phase cells (*p* < 10^–5^). We also observed that the exponential phase cells are localized to the voids of the fibrin network (Figure 3D) rather than bound to the fibrin fibers as seen for stationary phase cells (Figure 3E).

## Discussion

Bacteria in biofilms are phenotypically different than rapidly growing bacteria. More specifically, planktonic bacteria in a mid-exponential growth phase are more susceptible to antibiotics and bacteriophage therapy as compared to both planktonic bacteria in a stationary growth phase and bacteria in a biofilm (Spoering and Lewis, 2001; Keren et al., 2004; Cerca et al., 2007). The number of *S. epidermidis* cells able to survive antibiotic therapy increases with bacterial concentration in both planktonic and biofilm cultures (Shapiro et al., 2011). Additionally, bacterial cells are killed uniformly throughout the biofilm (Shapiro et al., 2011) which suggests that this mechanism of resilience is not necessarily impaired penetrance or inactivation of antibiotic through a biofilm, but rather some other difference in the bacteria related to its growth phase. There are likely many other phenotypic differences associated with growth phase that aid in survival and virulence.

This study demonstrates the growth phase dependent interactions between *S. epidermidis* and human fibrinogen. We further elucidated the resulting growth phase dependent effect of *S. epidermidis* on fibrin network formation, mechanics, and structure. Specifically, *S. epidermidis* in its stationary phase–versus exponential phase–expresses more *sdrG*, has increased affinity for fibrinogen, and adheres more frequently and for a longer duration to a fibrinogen coated surface. Additionally, presence of stationary phase *S. epidermidis* during thrombin-catalyzed polymerization of fibrinogen generates a heterogeneous fibrin network structure that is four times stiffer than pure fibrin in its absence. This effect is not observed if exponential phase *S. epidermidis* is present. Here we consider the possible explanations for the correlation between adhesion to fibrinogen and the resulting influence on fibrin clot formation, implications for virulence, and increased risk of embolism as a potential virulence factor for biofilm infected indwelling medical devices.

Previous studies have shown that bacterial infections increase the risk of thromboembolic disease (Raad et al., 1994; Grau et al., 1995; Samama, 2000; Brackman et al., 2013; Dalager-Pedersen et al., 2014) and specifically that *S. epidermidis* infected thrombi are less stable and more prone to rupture (Ma et al., 2017). Biofilms provide protection from host defenses and bacteria in biofilm show reduced susceptibility to antibiotics making biofilm infections difficult to clear (Shapiro et al., 2011). This study provides evidence that phase-dependent changes in gene expression, fibrinogen binding, and changes to clot mechanics affords the bacteria additional virulence in its biofilm-like state. The increased binding to fibrinogen coated surfaces implies additional opportunity to establish infection of indwelling medical devices and materials as well as natural surfaces such as cardiac valves. Indeed, fibrinogen has been shown to form a bridge, binding *S. aureus* with the endothelium (Cheung et al., 1991). In our previous work (Ma et al., 2017), the heterogeneous fibrin network structure observed in Figure 3E was linked to the higher stiffness (*G*′) *via* microrheology. The resulting disorganized fibrin network microstructure leads to instability and ultimately rupture.

**FIGURE 4:**
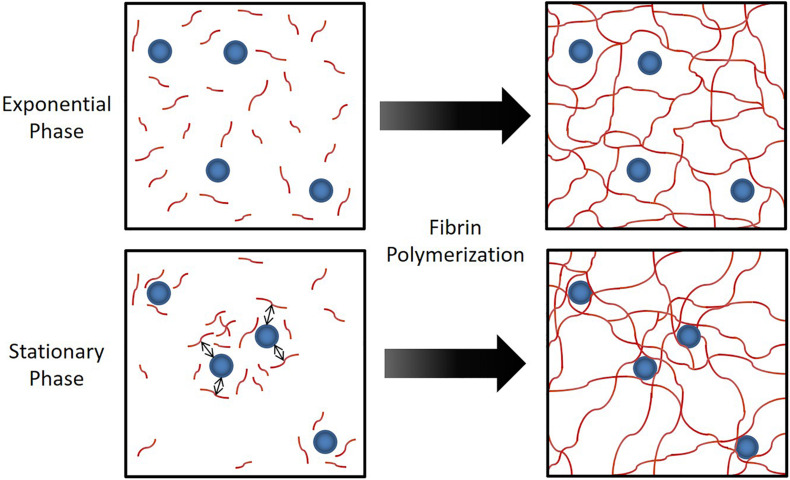
Hypothesized mechanism for the observed phenomenon in Figure 3. When *S. epidermidis* cells (blue circles) have sufficient affinity for fibrinogen (red lines), the attraction between cells and fibrinogen generate a heterogeneous fibrinogen distribution that results in a heterogeneous fibrin network structure. This has profound implications on fibrin network elasticity and ultimately potential for embolization.

In Figure 4, we conceptualize the idea of how strong attraction between cells and fibrinogen could lead to the formation of a heterogeneous fibrin network structure. With cells that strongly attract fibrinogen, the originally uniform distribution of fibrinogen molecules can shift to an increased concentration around cells, thus a transition to a heterogeneous distribution, which would ultimately lead to a heterogeneous fibrin network structure. Alternatively, a similar bacterial-thrombin interaction could result in a heterogeneous distribution of thrombin leading to a heterogeneous fibrin polymerization pattern. In addition to altered thrombus structure and mechanics, up-regulation of *sspA* (Table 1), a serine protease which cleaves fibrinogen, may offer an additional means of dispersion *via* thromboembolism and further disseminate infection. However, the exact contribution of this protease to clot rupture was not specifically evaluated in this study and is the subject of future work.

An increase in *sdrG* expression in stationary phase *S. epidermidis* cells may explain the observed phenotypic differences in affinity, deposition, and residence time (Figures 1, 2). However, the change in expression was quite modest (∼twofold) and other factors are likely in play. Bacteria can also bind non-specifically to surfaces as well as other molecules *via* electrostatic or Van der Waals forces (McGuffie et al., 2016; Ma et al., 2017). In addition, in this experimental system, thrombin, a positively charged enzyme that cleaves fibrinogen and initiates the fibrin polymerization, is also present and can be attracted to the negatively-charged cell wall surface. The surface charge of *S. epidermidis* has a distinct dependence on growth phase and nutrient environment (Gilbert et al., 1991).

We attempted to clarify the role of *SdrG* in the phase dependent effects observed in this study by evaluating the well characterized wild type HB *S. epidermidis* strain [*SdrG*^(+)^] and an isogenic *SdrG* disruption mutant [*SdrG*^(–)^] (details in Supplementary Figure 2). However, the parent strain did not have the same phase dependent response as seen for RP62A (Supplementary Figure 2). There was an expected trend toward decreased affinity for fibrinogen in the *SdrG* mutant although this did not reach significance for the small *N* = 3 sample. In light of the known strain-dependent differences in fibrinogen binding (Vanzieleghem et al., 2015), and lack of phase dependent response in the wild type strain, definitive role for *SdrG* in phase dependent binding could not be determined. That said, our prior work suggests that nonspecific binding through electrostatic interactions are sufficient for particle localization on to a fibrin network (Ma et al., 2017). This is also supported by the fact that lack of *SdrG* does not abolish but merely reduces binding to fibrinogen (Vanzieleghem et al., 2015).

It is worth noting that affinity to fibrinogen is not absent for exponential phase cells. This may be due to a baseline amount of *sdrG*-fibrinogen specific binding but a proportionally larger amount of non-specific binding in exponential phase. This change in proportion from non-specific to specific binding of fibrinogen may explain why stationary phase cells, but not exponential phase cells, affect clot formation and structure despite the amount of adhesion observed in the flow cytometry and cell deposition studies for exponential phase. We therefore speculate that the phase-dependent increase in *sdrG* may contribute not only to a higher frequency of adherence, but a stronger binding that leads to the heterogeneous fibrin distribution as depicted in Figure 4. This is supported by the fact that not only do we see increased deposition of stationary phase but longer duration of binding under shear conditions (Figure 2H), consistent with the force/shear loading enhanced binding observed in the literature (Weaver et al., 2011; Milles et al., 2018).

These results allow for speculation on the mechanistic implications for medical device infection, the potential for disseminated infection, and the correlation with thromboembolic events. Specifically, high baseline fibrinogen binding allows planktonic *S. epidermidis* in the bloodstream to adhere to fibrinogen/fibrin coated materials. As cell density increases this bond is strengthened consistent with observed maturation phase of the biofilm life cycle (Paharik and Horswill, 2016). Ongoing fibrin network formation is altered by the presence of *S. epidermidis* leading to a stiffer clot with increased potential for rupture and embolization (Ma et al., 2017). Simultaneous production of proteases like *SspA* further facilitate dissemination of cells back to the bloodstream. The affinity of free floating stationary phase of *S. epidermidis* for fibrinogen is correlated with decreased opsonization and phagocytosis by macrophages (Rennermalm et al., 2004). This provides a means for immune evasion as well as opportunity to aggregate *via* platelets (Brennan et al., 2009). Finally, the potential for adhesion to a new surface location is facilitated and the process starts anew.

## Conclusion

In conclusion, this study demonstrates the growth phase dependent interaction between *S. epidermidis* and fibrinogen and the resulting effect of *S. epidermidis* on fibrin formation, structure and mechanics. It contributes to the current understanding of growth phase dependent regulation of *S. epidermidis* virulence factors and the association between bacterial infection and thrombosis. It also provides fodder for investigation of the mechanisms of and pharmaceutical solutions to infection-induced thromboembolism.

## Data Availability Statement

The raw data supporting the conclusions of this article will be made available by the authors, without undue reservation.

## Author Contributions

CV, TM, MS, and JV conceptualized the study. CV, TM, JS, UK, CA, and EM-N performed the experiments and generated the data. Formal analysis was performed by CV, JM, EM-N, and JV. MS and JV provide resources, funding, and supervision. JV provided project administration. All authors contributed to writing, reviewing, and editing.

## Conflict of Interest

The authors declare that the research was conducted in the absence of any commercial or financial relationships that could be construed as a potential conflict of interest.
