# Supplementary material for: Staphylococcus epidermidis Has Growth Phase Dependent Affinity for Fibrinogen and Resulting Fibrin Clot Elasticity
Source: Front Microbiol. 2021 Jun 16;12:649534. doi: 10.3389/fmicb.2021.649534 (PMC8241941; doi:10.3389/fmicb.2021.649534)
Supplement: Supplementary file 1 [file Data_Sheet_1.PDF]

Supplementary information for

Staphylococcus epidermidis has growth phase dependent affinity for fibrinogen and resulting fibrin clot elasticity

Carolyn Vitale, Tianhui Maria Ma, Janice Sim, Christopher Altheim, Erika Martinez-Nieves, Usha Kadiyala, Michael J. Solomon, and J. Scott VanEpps

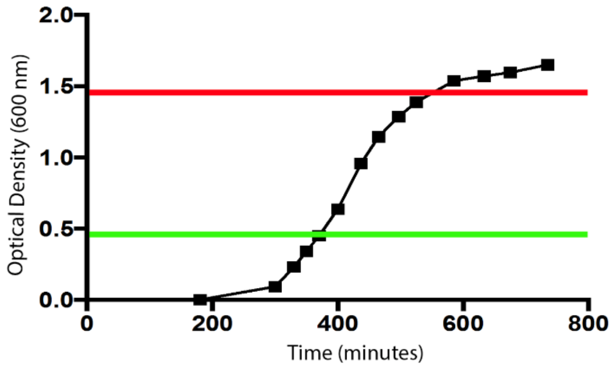

Supplementary Figure S1: Growth curve, determined by optical density at 600 nm, of *S. epidermidis*. For the purposes of this study exponential phase cells had an optical density of 0.4±0.1 (green line) and stationary phase cells had an optical density of 1.4±0.05 (red line).

Supplementary Table 1: PCR oligonucleotides (5'→3')

Gene	Forward primer	Reverse primer	Probe	Amplicon
icaA	AGGGAATCAAACAAGCATCT	TGTCCATTAGGTGCATAAGC	CTGACACTGTCATTGATGACGATGC	142
aap	TTAGGATTGAGTCCAACTGATAG	GACTGCTTTAGGAGTGTATGT	CAAGTGGCGTTATGAGAGCTGATTTAGATG	141
atlE	ATTATTAGTTCCTCCTGATG	GCTATGCAGAATTATATGAC	AGGTGCTACTTGCTTCGTTT	125
sdrG	CGGACTGCTAACCTTCAAAGTA	ATAAAGTCCCTTACCGCTACTT	ACCCTCTTCGTTATTCAGCCAAAGAAACA	135
sdrF	TTCTATCCAACAAGGTAAAC	GCTGATTCTAATTGATTGTC	CAGTAGGTACAGCTTCAATACTC	139
sspA	CCAATATAGACAACTTTACC	GCAACTATAAGTAGCAATAC	CAGGGTAACCAGTAACAGTGATG	119
icaR	TGCTTCTGGAGCACTAGATAA	TGCTTAGGAACCATGATGATAA	AAACTGGTAAAGTCCGTCAATGGAA	125
16s rRNA	GTAAACGATGAGTGCTAAGTGT	CCGTCAATTCCTTTGAGTTTCA	CCGCCTGGGGAGTACGACCGCAA	116

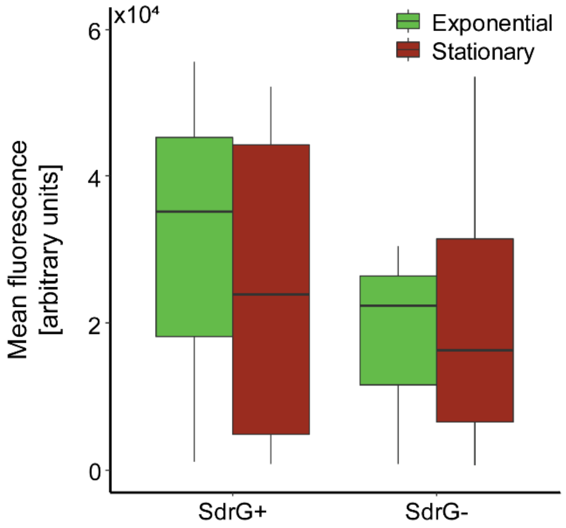

Supplementary Figure S2: The wild type *S. epidermidis* HB strain (SdrG+) and the mutant SdrG– strain in which the *sdrG* gene was disrupted by integration of the pG+Host9*fbe* plasmid were graciously provided by Professor Timothy J. Foster(Herman et al., 2014). These strains were grown under the same conditions and processed for flow cytometry as we described in the methods for RP62A. Standard box (IQR) and whiskers (1.5*IQR) plot of mean fluorescence of for the SdrG+ and SdrG– strains are shown for the exponential and stationary phase. Kruskal-Wallis test was used as a nonparametric analog to ANOVA to compare differences associated with growth phase and strain (N=3). No significant differences were observed although a trend toward decrease in fibrinogen binding was observed for the SdrG– with respect to SdrG+.

References:

Herman, P., El-Kirat-Chatel, S., Beaussart, A., Geoghegan, J.A., Foster, T.J., and Dufrêne, Y.F. (2014). The binding force of the staphylococcal adhesin SdrG is remarkably strong. *Molecular Microbiology* 93(2), 356-368. doi: https://doi.org/10.1111/mmi.12663.
